# Pemafibrate prevents choroidal neovascularization in a mouse model of neovascular age-related macular degeneration

**DOI:** 10.7717/peerj.14611

**Published:** 2023-01-10

**Authors:** Deokho Lee, Ayaka Nakai, Yukihiro Miwa, Kazuno Negishi, Yohei Tomita, Toshihide Kurihara

**Affiliations:** 1 Laboratory of Photobiology, Keio University School of Medicine, Tokyo, Japan; 2 Ophthalmology, Keio University School of Medicine, Tokyo, Japan; 3 Ophthalmology, Nihon University School of Medicine, Tokyo, Japan; 4 Aichi Animal Eye Clinics, Aichi, Japan

**Keywords:** Neovascularization, Pemafibrate, Choroid, Eye

## Abstract

**Background:**

Pathological choroidal neovascularization (CNV) is one of the major causes of visual impairment in neovascular age-related macular degeneration (AMD). CNV has been suppressed by using anti-vascular endothelial growth factor (VEGF) antibodies. However, some clinical cases have demonstrated the failure of anti-VEGF therapies. Furthermore, anti-VEGF agents might induce the development of ocular atrophy. Recently, peroxisome proliferator-activated receptor alpha (PPAR*α*) activation using pemafibrate treatment was suggested as one of the promising therapeutic targets in the prevention of ocular ischemia. However, the preventive role of pemafibrate remains unclear in CNV. We aimed to examine the preventive role of pemafibrate on laser-induced pathological CNV.

**Methods:**

Adult male C57BL/6 mice were orally supplied pemafibrate (0.5 mg/kg) for four days, followed by laser irradiation. Then, pemafibrate was consecutively given to mice with the same condition. CNV was visualized with isolectin-IB4. The eye (retina and/or retinal pigment epithelium [RPE]-choroid), liver, and serum were used for biomolecular analyses.

**Results:**

We found that pemafibrate administration suppressed CNV volumes. Pemafibrate administration activated PPAR*α* downstream genes in the liver and eye (especially, RPE-choroid). Furthermore, pemafibrate administration elevated serum fibroblast growth factor 21 levels and reduced serum levels of triglycerides.

**Conclusions:**

Our data suggest a promising pemafibrate therapy for suppressing CNV in AMD.

## Introduction

Neovascular age-related macular degeneration (AMD) involves choroidal neovascularization (CNV), which is one of the leading causes of blindness globally. For the current treatment, anti-vascular endothelial growth factor (VEGF) antibodies were used to suppress pathological CNV, in that VEGF plays a central role in developing pathological CNV (Solomon et al., 2019). Although anti-VEGF therapies are effective for the majority of neovascular subjects, some clinical cases have demonstrated the failure of anti-VEGF therapies, suggesting VEGF may not be the only target for CNV formation (Mettu, Allingham & Cousins, 2021). Moreover, anti-VEGF antibodies may induce the development of atrophy in the eye (Eng et al., 2020; Foss et al., 2022). As the delivery method of anti-VEGF antibodies is invasive to the patients (Nikkhah et al., 2018) and patients always need to go to clinics for the treatment, more advanced patient friendly approaches might be also desirable. In this regard, various alternative therapies have been searched and tested in a pre-clinical stage. Nonetheless, no effective drug has been developed and found yet.

Peroxisome proliferator-activated receptor alpha (PPAR *α*) is one of the nuclear receptor proteins that promote ligand-dependent transcription of various genes involved in energy production, lipid metabolism, and inflammatory process (Bougarne et al., 2018). In addition to PPAR *α*, two other isotypes, such as PPAR *β*/*δ* and PPAR *γ*, also exist with displaying isoform-specific functions in cells (Christofides et al., 2021). PPAR *α* activation by pemafibrate (a selective PPAR *α* modulator; SPPARM *α*) has been reported to be beneficial in enhancing metabolic dysregulation in patients with dyslipidemia (Ida, Kaneko & Murata, 2019). Based on a previous literature, pemafibrate could decrease serum triglyceride levels and increased serum high-density lipoprotein cholesterol levels more than other PPAR *α* agonists, which implies that pemafibrate may have a better potency and selectivity for the activation of PPAR *α* than other PPAR *α* agonists (Tomita et al., 2020b). Furthermore, side effects of pemafibrate have been reported less than those of other PPAR *α* agonists (especially, fenofibrate). It was explained with their structural differences in that pemafibrate contains not only the carboxylic acid group but also the phenoxy alkyl group and 2-aminobenzoxazolic group making the ligand/receptor binding fit well (Tomita et al., 2020b).

In addition to the positive outcome for dyslipidemia, recently, PPAR *α* activation by pemafibrate treatment was also suggested as one of the promising therapeutic targets in the prevention of ocular ischemic diseases (Lee et al., 2021b). Our previous reports demonstrated that oral administration of pemafibrate could prevent pathological retinal neovascularization *via* decreasing *Vegf* mRNA expression and hypoxia-inducible factor-1 *α* (HIF-1 *α*; one of the master regulatory transcription factors for VEGF in the body including the eye (Ahluwalia & Tarnawski, 2012; Lee et al., 2022a)) protein immunoreactivity in a murine model of oxygen-induced retinopathy (Tomita et al., 2019). Furthermore, pemafibrate administration could suppress retinal dysfunction and/or pathological gliosis *via* modulating multiple therapeutic gene expressions in a murine model of unilateral common carotid artery occlusion-induced ocular ischemia (Lee et al., 2021c). Its administration could also increase retinal synaptophysin expression (one of the crucial integral membrane proteins regulating endocytosis for synaptic vesicles (Kwon & Chapman, 2011) and suppress retinal dysfunction in a murine model of streptozotocin-induced diabetic retinopathy (Tomita et al., 2020a). Recently, oral administration of pemafibrate could reduce retinal ganglion cell death and suppress retinal dysfunction through modulating various therapeutic gene expressions (anti-oxidant (Loboda et al., 2016) and anti-inflammatory pathways (Netea et al., 2008; Rashid, Akhtar-Schaefer & Langmann, 2019)) in a murine model of transient high intraocular pressure-associated retinal ischemia-reperfusion injury (Lee et al., 2022b). Other groups’ reports demonstrated that pemafibrate administration could protect against N-methyl-D-aspartate (NMDA)-induced rat retinal ganglion cell death *via* inhibition of phosphorylated c-Jun expression in the eye (Fujita et al., 2021), or reduce retinal inflammation (vascular leakage or leukostasis) *via* upregulating thrombomodulin expression (one of the important transmembrane factors placed on the surface of endothelial cells (Sadler, 1997) in a rat model of streptozotocin-induced diabetic retinopathy (Shiono et al., 2020). Taken together, although the therapeutic role of pemafibrate on retinal function, protection, and neovascularization has been suggested, the preventive role of pemafibrate remains unclear in pathological CNV.

Thus, in this current study, we aimed to first investigate the preventive effects of pemafibrate in a mouse model of laser-induced CNV, one of the mouse models of neovascular AMD.

## Materials & Methods

### Animals and laser-induced choroidal neovascularization (CNV)

Male adult mice (6–8 weeks old C57BL/6, *n* = 48) were received from CLEA Japan (Tokyo, Japan). Mice were maintained in a temperature (24 ± 1 °C)-controlled environment (6 mice per cage) under a 12 h light-dark cycle. The mice were subjected to general randomization and 1 week acclimatization. Food and water were freely supplied to mice without any restriction. Any pathologic sign of diseases were generally checked during the whole experimental period. All mouse procedures adhered to the Ethics Committee on Animal Research of the Keio University School of Medicine (#16017). The ARVO Statement for the Use of Animals in Ophthalmic and Vision Research, and the international standards of animal care and use, Animal Research: Reporting *in vivo* Experiments guidelines were further followed.

Laser-induced CNV was developed, as described in our previous research (Ibuki et al., 2020). Tropicamide and phenylephrine (Santen Pharmaceutical, Osaka, Japan) was generally applied for mouse pupil dilation. A mixture of midazolam (40 µg/100 µL, Sandoz, Tokyo, Japan), medetomidine (7.5 µg/100 µL, Orion, Espoo, Finland), and butorphanol tartrate (50 µg/100 µL, Meiji Seika Pharma, Tokyo, Japan), simply known as ‘MMB’, was applied for general mouse anesthesia. After pupil dilation and anesthesia (within 5 min of MMB injection), the mice’ eyes were gently placed with a contact lens, and four CNV spots (532 nm argon laser, 100 mw, 100 ms, 75 µm) were made between the ocular vessels at 2-disc diameters from the mouse’s optic nerve head. Air bubbles were generally indicated as a positive sign of disruption of the Bruch’s membrane by laser irradiation. CNV spots lacking air bubbles or having extensive hemorrhage were not included for further data analyses. After euthanasia (using a combination of 3x of MMB injection to mice), flat-mounted choroidal complexes were made from the mouse eyes by micro-scissors and incubated in isolectin IB4 solution (IB4 from *Griffonia simplicifolia*, Invitrogen, Carlsbad, CA, USA). CNV was detected and visualized by LSM710 microscope (Carl Zeiss, Jena, Germany) and the CNV volumes were determined using the Imaris software (Bitplane, Zurich, Switzerland).

During the experimental period in which disease signs (including hunched posture, lethargy, lack of food intake, or unexpected infection) were detected in experimental models, a combination of 3x of MMB was given to mice for deep anesthesia, and then mice were euthanized (Lee et al., 2021a; Xie et al., 2021).

### Quantitative PCR

A series of steps for quantitative PCR (qPCR) were conducted using RNA extraction, cDNA synthesis, and qPCR kits (RNeasy Plus Mini Kits, Qiagen, Venlo, The Netherlands; ReverTra Ace qPCR RT Master Mix, TOYOBO, Osaka, Japan; THUNDERBIRD SYBR qPCR Mix, TOYOBO, Osaka, Japan, respectively), as previously described (Lee et al., 2022b; Lee et al., 2021c; Tomita et al., 2019). Briefly, mouse tissues were dissolved in TRI reagent solution. After 3 min of incubation at room temperature, chloroform (a third of the TRI solution) was added to each sample. After gentle mixing followed by spin-down, each sample’s solution was transferred to Econospin columns for RNA collection. The columns were washed with buffer RWT and RPE (Qiagen, Hilden, Germany) two times. ND-2000 spectrophotometer (Thermo Fisher Scientific, Waltham, MA, USA) was used for checking each sample’s quality and quantity. Then, for cDNA synthesis, its sample (500 ng RNA) was transferred to PCR tubes with cDNA synthesis solutions above and synthesized by following the manufacturer’s instructions. The Step One Plus Real-Time PCR machine (Applied Biosystems, Waltham, MA, USA) was used for qPCR analyses with SYBR qPCR mixture. The primers used in the current study are the same as those in our previous reports (Table 1) (Lee et al., 2022b; Lee et al., 2021c; Tomita et al., 2019). The fold alteration between different transcripts’ levels was calculated by the general Δ ΔCT method.

**Table 1:** Primer list.

Name	Direction	Sequence (5′→ 3′)	Accession number
*Hprt*	Forward	TCAGTCAACGGGGGACATAAA	NM_013556.2
	Reverse	GGGGCTGTACTGCTTAACCAG	
*Fabp4*	Forward	CCGCAGACGACAGGA	NM_024406.3
	Reverse	CTCATGCCCTTTCATAAACT	
*Fgf21*	Forward	AACAGCCATTCACTTTGCCTGAGC	NM_020013.4
	Reverse	GGCAGCTGGAATTGTGTTCTGACT	
*Vldlr*	Forward	GAGCCCCTGAAGGAATGCC	NM_001161420.1
	Reverse	CCTATAACTAGGTCTTTGCAGATATGG	
*Acox1*	Forward	TCTTCTTGAGACAGGGCCCAG	AF006688.1
	Reverse	GTTCCGACTAGCCAGGCATG	
*Iba1*	Forward	CAGACTGCCAGCCTAAGACA	NM_001361501.1
	Reverse	AGGAATTGCTTGTTGATCCC	

### Serum assays

When it comes to mouse serum assays, mouse blood was freshly collected from the heart of each mouse. After 15 min of incubation at room temperature, serum samples were collected from each blood sample by centrifugation at 4 degree. Serum samples were placed on ice and immediately subjected to the further serum assays. Concentrations of serum fibroblast growth factor 21 (FGF21) and triglycerides (TG) were determined using FGF21 ELISA kits (Cat #RD291108200R; BioVendor Laboratory Medicine, Brno, Czech Republic) and TG kits (Cat #STA-396; Cell Biolabs, Inc., San Diego, CA, USA), as described in our previous studies (Lee et al., 2022b; Lee et al., 2021c). Serum samples were diluted to 1/2 for the FGF21 assay, while the samples were diluted to 1/10 for the TG assay. For the whole procedures, we directly followed the manufacturer’s instructions.

### Immunohistochemistry

Immunohistochemistry (IHC) was conducted as described in our previous reports (Lee et al., 2022b; Lee et al., 2021c). A total of 4% Paraformaldehyde (PFA) was used to fix the mouse eyeballs. After 3 h of incubation at 4 degree, the PFA-fixed eyeballs were transferred to Petri dish with cold PBS, and flat-mounted using micro-scissors. Flat mounted-samples were stained by a primary antibody (IBA1, 1:400, Cat #019-19741; Wako Chemicals, Richmond, VA, USA) for 24 h. After washing with cold PBS containing 0.3% Triton X three times, the samples were further incubated by a fluorescence-conjugated secondary antibody (1:400; Thermo Fisher Scientific, Waltham, MA, USA) for 2 h. After washing with the same solution above three times, samples were gently mounted with a cover glass. After drying for a few minutes, fluorescence signals in the samples were detected by LSM710 microscope.

### Statistical analysis

Statistical significance was set using a Student’s *t*-test or ANOVA followed by a Bonferroni post hoc test. Mean or mean ± standard deviation was selected for general figuration. *p* < 0.05: statistically significant.

## Results

### Oral administration of pemafibrate suppresses laser-induced choroidal neovascularization (CNV) in a mouse model of neovascular age-related macular degeneration (AMD)

**Figure 1:**
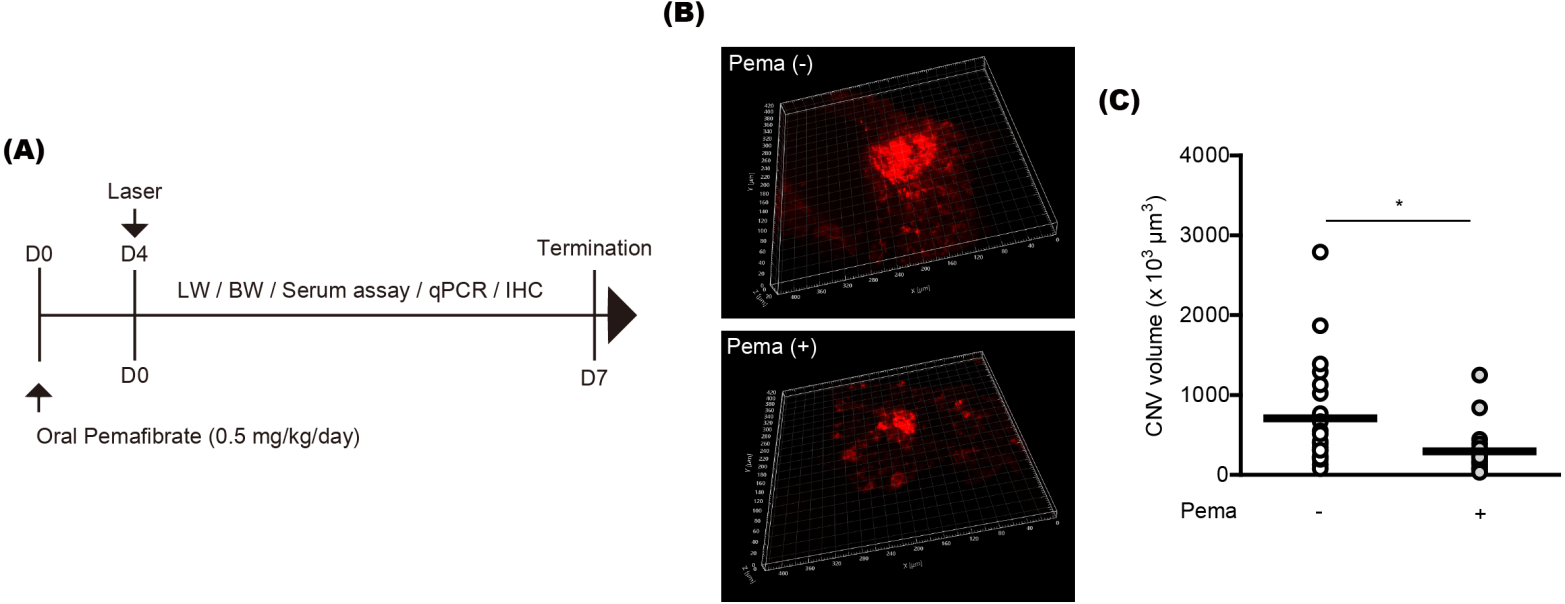
Suppression of choroidal neovascularization (CNV) by oral pemafibrate administration in a mouse model of neovascular age-related macular degeneration (AMD). (A) Schematic illustration for the whole experiment. D, day; LW, liver weight; BW, body weight; qPCR, quantitative PCR; IHC, immunohistochemistry. (B and C) Representative images of CNV stained by isolectin IB4 and quantitative analyses ( *n* = 18–21 per group) demonstrated that the volume of CNV was dramatically reduced by pemafibrate administration. ∗*p* < 0.05. Graphs were presented as mean. The data were analyzed using Student’s *t*-test (two-way). Pema: pemafibrate.

To examine the preventive effect of pemafibrate on CNV formation, mice were orally administered pemafibrate four days before laser irradiation (Fig. 1A). Oral administration was conducted using a general mouse gavage. After the irradiation, mice were continuously administered pemafibrate every day until the end of the experiment. The concentration of pemafibrate (0.5 mg/kg/day) was determined based on our previous papers (Lee et al., 2022b; Lee et al., 2021c). Reductions in CNV volumes were significantly detected in pemafibrate-administered mice 7 days after the irradiation (Figs. 1B and 1C).

**Figure 2:**
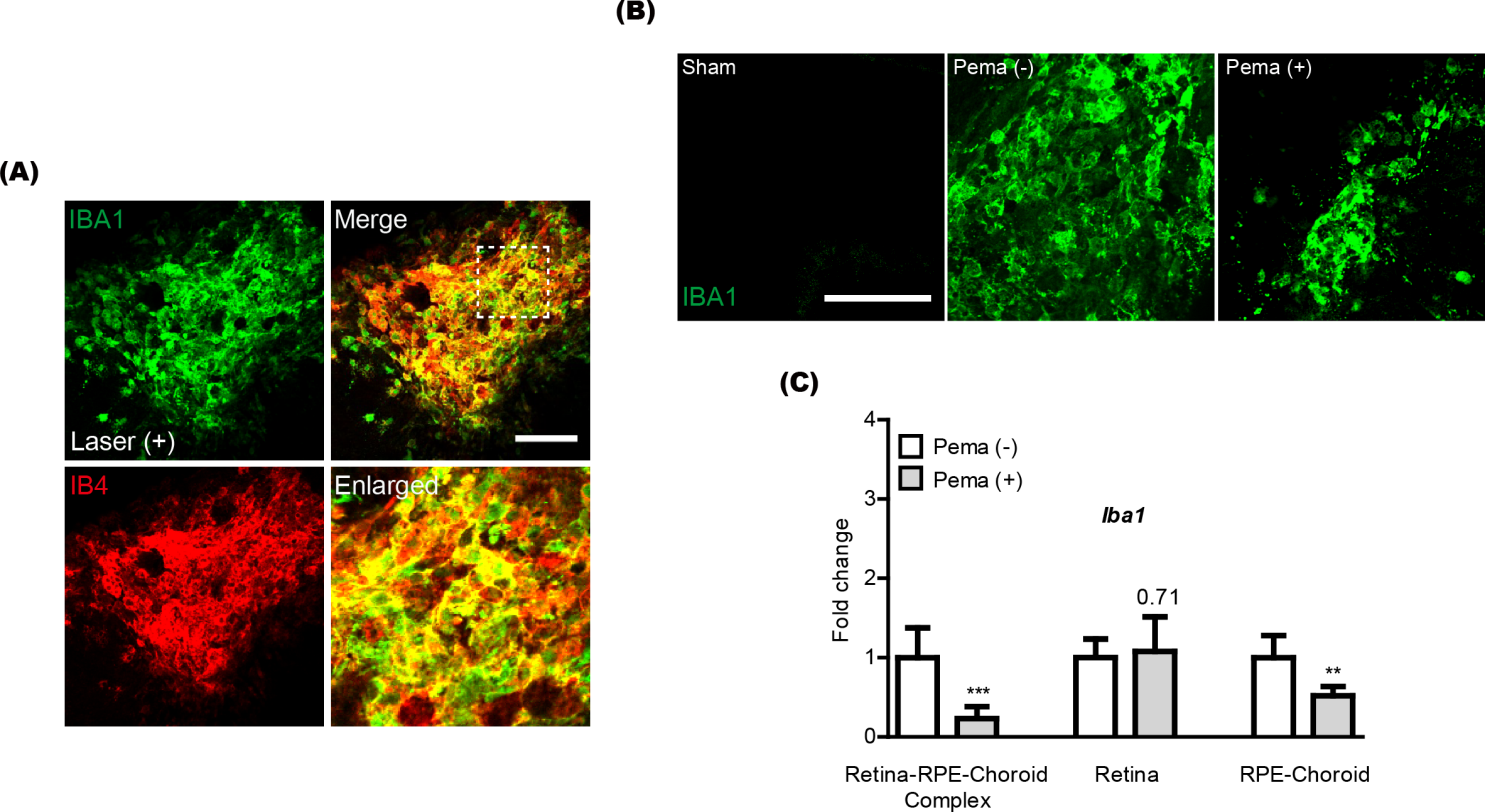
Reduction of activated microglia by oral pemafibrate administration in a mouse model of neovascular age-related macular degeneration (AMD). (A and B) Representative images of CNV co-stained by isolectin IB4 and IBA1 (one of the microglial markers) 7 days after laser irradiation. Representative images of IBA1 staining in sham-operated, laser-irradiated, and laser-irradiated pemafibrate-administered CNV 7 days after laser irradiation. Scale bar: 100 µm. (C) Quantitative analyses (*n* = 6 per group) indicated that *Iba1* mRNA expression decreased in the retina-retinal pigment epithelium (RPE)-choroid complex and RPE-choroid complex by oral pemafibrate administration 7 days after laser irradiation. Graphs were depicted as mean ± standard deviation. ^∗∗^*p* < 0.01, ^∗∗∗^*p* < 0.001. The data were analyzed using Student’s *t*-test (two-way). Pema: pemafibrate.

Previously, activated microglia has been reported to co-label with CNV (Wang et al., 2021). Thus, we also checked this aspect in our current system and found that IBA1 staining (a microglial marker) was co-labeled with IB4 staining in CNV (Fig. 2A). Reductions in IBA1 and *Iba1* mRNA expression were clearly detected in the pemafibrate-administered CNV and retina-RPE-choroid complex, respectively (Figs. 2B and 2C). We further found reduced *Iba1* mRNA expression in the pemafibrate-administered RPE-choroid complex (Fig. 2C). There was no significant change in *Iba1* mRNA expression in the retina between the groups.

### Oral administration of pemafibrate systemically activates peroxisome proliferator-activated receptor alpha (PPAR *α*) target genes in a mouse model of neovascular age-related macular degeneration (AMD)

**Figure 3:**
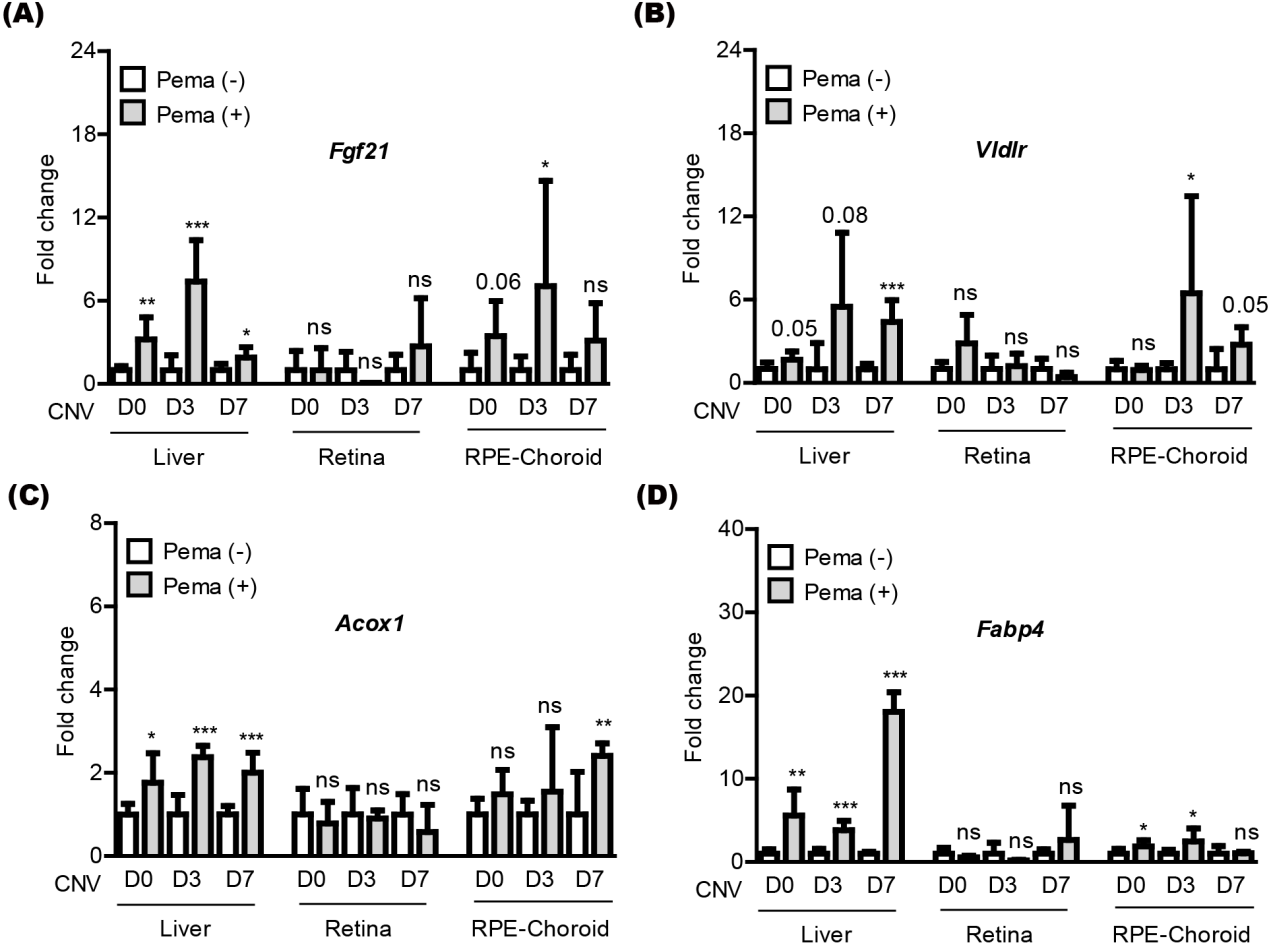
Screening of PPAR *α* downstream gene expressions in the liver and eye by oral pemafibrate administration in a mouse model of neovascular age-related macular degeneration (AMD). (A–D) Quantitative analyses (*n* = 6–8 per group) demonstrated that pemafibrate administration did not change PPAR *α* downstream gene expressions in the retina, while its administration increased PPAR *α* downstream gene expressions in the liver or retinal pigment epithelium (RPE)-choroid on the day of laser irradiation (D0) and 3 and 7 days after the irradiation (D3 and D7). Graphs were depicted as mean ± standard deviation. ^∗^*p* < 0.05, ^∗∗^*p* < 0.01, ^∗∗∗^*p* < 0.001. The data were analyzed using Student’s *t*-test (two-way). Pema: pemafibrate.

We attempted to examine pemafibrate-induced PPAR *α* activation in the mouse body (especially, the liver, retina, and RPE-choroid) (Fig. 3). The eye was our target of interest, while the liver has been widely known as the main PPAR *α* activation site by pemafibrate treatment (Lee et al., 2022b; Lee et al., 2021c). PPAR *α* target genes (*Fgf21*, *Vldlr*, *Acox1*, and *Fabp4*) were selected, as those genes have been generally known activated by treatment of PPAR *α* agonists (Lee et al., 2022b; Lee et al., 2021c). We examined those gene expressions in tissues under our current experimental condition (Figs. 3A–3D). We found that PPAR *α* downstream gene expressions were increased by oral administration of pemafibrate in the liver and RPE-choroid, not in the retina, although there were fluctuations in the RPE-choroid depending the genes.

**Figure 4:**
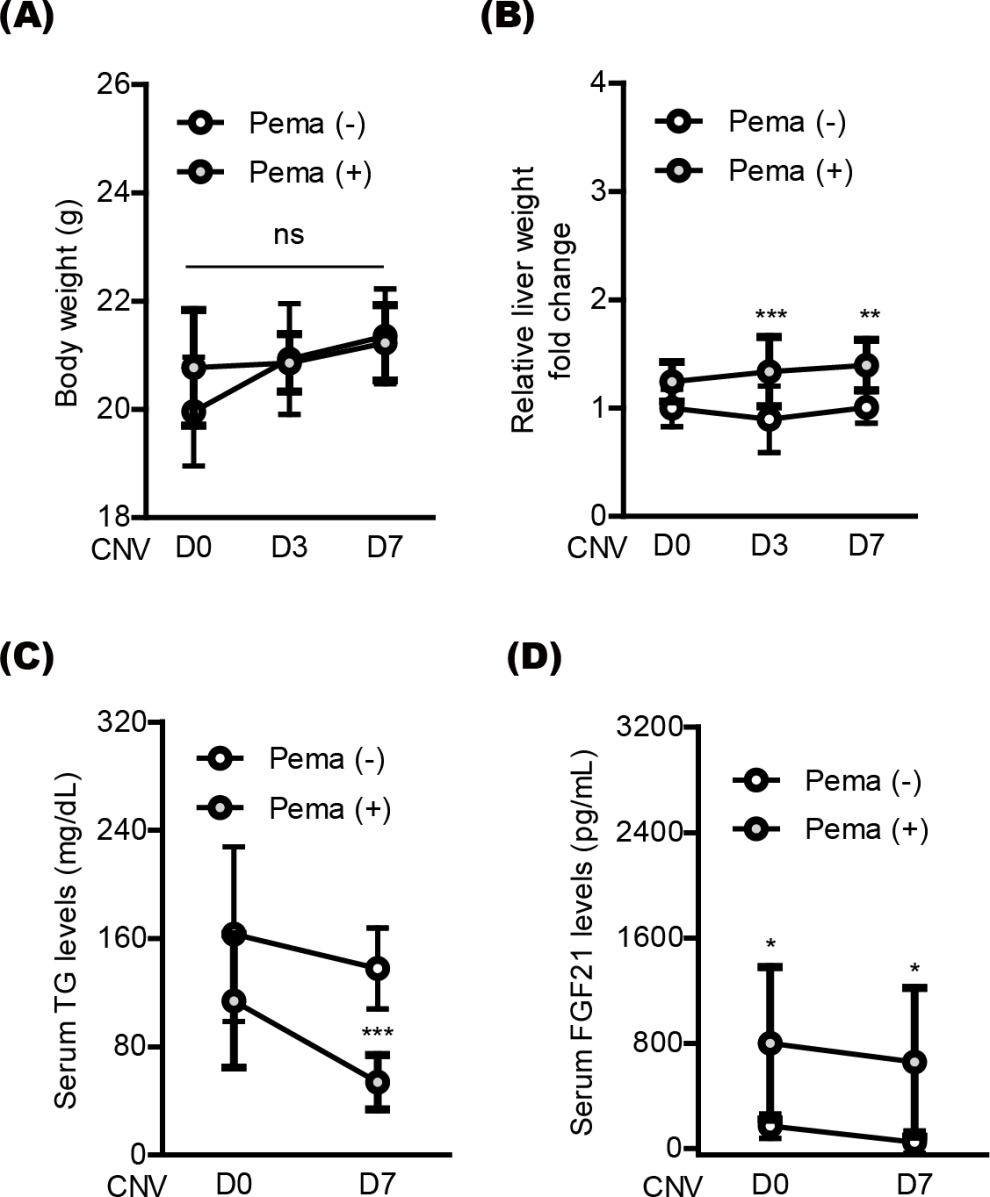
Screening of systemic factors by oral pemafibrate administration in a mouse model of neovascular age-related macular degeneration (AMD). (A and B) Quantitative analyses (*n* = 10 per group) indicated that the body weight was not changed by pemafibrate administration, while the liver weight significantly increased. (C) Serum triglyceride (TG) levels significantly decreased by pemafibrate administration from day 0 (D0) to day 7 (D7) after laser irradiation (*n* = 6–10 per group). (D) Increased serum levels of FGF21 were maintained by oral pemafibrate administration (*n* = 6–7 per group). Graphs were depicted as mean ± standard deviation. ^∗^*p* < 0.05, ^∗∗^*p* < 0.01, ^∗∗∗^*p* < 0.001. The data were analyzed using two-way ANOVA followed by a Bonferroni post hoc test. Pema: pemafibrate.

Next, we moved to investigate systemic factors in mice after oral pemafibrate administration. There was no significant alteration in the body weight by oral pemafibrate administration during the experimental observation period from day 0 (D0) to day 7 (D7) (Figs. 4A and 4B), while the relative liver weight was increased by oral pemafibrate administration, which was similar to that reported in our previous pemafibrate papers (Lee et al., 2022b; Lee et al., 2021c). We further found that TG levels significantly decreased by oral pemafibrate administration (Fig. 4C), while FGF21 levels dramatically increased by its administration (Fig. 4D). Elevated serum levels of FGF21 were maintained for 7 days after laser irradiation (D7, the termination day of our current experimental observation).

## Discussion

In our current study, oral pemafibrate administration suppressed pathological CNV along with reductions in ocular microglial activation in a murine model of neovascular AMD. A significant induction in PPAR *α* target gene expressions in the liver and eye (especially, RPE-choroid), a reduction in serum levels of TG, and an elevation in serum levels of FGF21 were detected after consecutive oral administration of pemafibrate. Previously, pemafibrate has been suggested as a promising drug to intervene in various pathological mechanisms in several experimental models of retinopathies (Lee et al., 2021b). However, we first reported the preventive role of pemafibrate in laser-induced pathological CNV formation in a murine model of neovascular AMD, which is the significance of our study.

The preventive roles of PPAR *α* activation on CNV formation have been reported. Zhao et al. (2018) demonstrated that fenofibrate treatment into the vitreous cavity could inhibit laser-induced CNV formation in Brown Norway rats. Gong et al. (2016) suggested that fenofibrate administration could reduce laser-induced CNV formation in mice. Huang et al. (2021) reported that topical treatment of the fenofibrate eye drop could suppress laser-induced CNV formation in mice. Pemafibrate might also show similar preventive effects to fenofibrate on CNV formation, in that pemafibrate is another PPAR *α* agonist similar to fenofibrate.

Retinal microglia has a significant role in ocular homeostasis. Microglial activation is known to be induced by various stresses, including hypoxic/ischemic injuries (Abcouwer et al., 2021; Weinstein, Koerner & Möller, 2010). Previous studies showed that microglia might be involved in inflammatory and neovascularization signaling pathways in laser-induced CNV (Hikage et al., 2021; Huang et al., 2013a; Kim et al., 2021). We also found that microglia resided around CNV in our current system. As pemafibrate administration could reduce *Iba1* expression in CNV, the preventive effects of pemafibrate on CNV formation could be explained by inhibiting activated microglia. Previously, pre-treatment of pemafibrate showed reductions in microglial activation *in vitro* (Ogawa et al., 2020). They further demonstrated that the other PPAR *α* agonist such as fenofibrate also suppressed microglial activation *in vitro*. Another previous study demonstrated that PPAR *α* activation using fenofibrate and GW7647 (both PPAR *α* agonists) treatment could suppress radiation-induced inflammatory responses (upregulation of *Tnf*- *α* and *Il*-1 *β* mRNA expressions) in activated microglial cells *in vitro* (Ramanan et al., 2008). In addition to the eye, previous studies on the central nervous system (especially, the brain) showed suppression of microglial activation by PPAR *α* agonists/ligands to improve various types of brain damages (Boujon et al., 2019; Grabacka et al., 2021; Ramanan et al., 2009; Wójtowicz et al., 2020; Zhou et al., 2022). Taken together, this can also support our current outcome *in vivo*.

FGF21 is regarded as a crucial regulator of energy metabolism. While FGF21 is mainly produced by the liver, it might also be expressed and secreted in other tissues including skeletal muscles and adipose (Staiger et al., 2017; Szczepańska & Gietka-Czernel, 2022; Tezze, Romanello & Sandri, 2019). In addition to the systemic energy metabolic role, FGF21 has been also suggested to have various therapeutic roles in the central nervous system including the eye (Sa-Nguanmoo, Chattipakorn & Chattipakorn, 2016; Yuan et al., 2021). Administration of a long-acting FGF21 analog, PF-05231023, exerted a suppressive effect on ocular neovascularization in mice (Fu et al., 2017). However, the administration of native FGF21 had lesser effects as it has a short half-life (0.4 h) (Fu et al., 2017; Huang et al., 2013b). In this regard, stable induction of circulating FGF21 by pemafibrate administration may effectively suppress CNV volumes during the CNV formation stages induced by laser irradiation. Furthermore, pemafibrate-induced FGF21 boosting (as one of the PPAR *α* target genes) in the eye (especially, RPE-choroid) may locally support suppressing CNV volumes in our present system. However, further studies are needed in this aspect.

Based on our current data, PPAR *α* target genes were upregulated by pemafibrate treatment. Those PPAR *α* target genes (*Fgf21*, *Vldlr*, *Acox1*, and *Fabp4*) were selected for the current experiment as they have been known as representative PPAR *α* downstream genes activated by PPAR *α* agonists. However, its knowledge has been mainly confirmed in the liver (Lee et al., 2022b; Lee et al., 2021c; Tomita et al., 2019). With this reason, we could reproduce its findings with pemafibrate treatment in the liver. When it comes to the eye, PPAR *α* target genes have not yet been actively studied, although those genes (*Fgf21*, *Vldlr*, *Acox1*, and *Fabp4*) have been generally used to screen for PPAR *α* activation. Furthermore, upregulation dynamics of those genes have not yet been reported. Taken together, more researches on PPAR *α* activation and its downstream gene upregulation in the eye are needed to be conducted.

In our current study, serum TG levels were decreased by oral pemafibrate administration. This reducing effect has been consistently detected in various experimental models from ours and others (Fruchart, Hermans & Fruchart-Najib, 2020; Tomita et al., 2020b). Its effect was already well confirmed in human studies on pemafibrate (Ida, Kaneko & Murata, 2019). High TG levels are considered as one of the risk parameters in the development or progression of human cardiovascular diseases with metabolic disorders (Budoff, 2016; Farnier et al., 2021; Peng et al., 2017). Although our murine CNV model may not have systemic metabolic injuries, high TG levels could worsen the progression of AMD in humans with metabolic disorders and diseases. Thus, pemafibrate treatment could be more powerful under disease conditions in humans. In this regard, a novel experimental murine model of laser-induced CNV with systemic metabolic dysregulation (using streptozotocin injection to induce diabetic conditions Furman, 2015) could be made, and the therapeutic effects of pemafibrate could be examined in that model, which could be further studied.

Pemafibrate has been well-treated for reducing triglycerides in clinic. Their safety and efficacy have been gradually stacked. Pemafibrate showed superior benefits-risk balance compared to conventional fibrates in human studies (Yamashita, Masuda & Matsuzawa, 2020). Experimental reports also supported the notion that pemafibrate treatment could be more beneficial than other conventional fibrates. In ophthalmic areas, positive effects of pemafibrate have also been gradually found (Lee et al., 2022c; Tomita et al., 2020b). In this regard, the use of pemafibrate could be promising in ophthalmic areas in terms of clinical trial time and safety concerns.

## Conclusions

In conclusion, although we need more evidence regarding CNV suppression by activating PPAR *α* in the liver and/or the eye, we suggest a promising pemafibrate therapy in laser-induced CNV, with enhancing liver function, controlling serum levels of FGF21 and TG, and suppressing retinal microglial activation.

##  Supplemental Information

10.7717/peerj.14611/supp-1 Supplemental Information 1 Raw data

## Additional Information and Declarations

### Data Availability

The following information was supplied regarding data availability: The raw data is available in the Supplementary Files.
